# Coronary artery bypass surgery plus medical therapy versus medical therapy alone for ischaemic heart disease: a protocol for a systematic review with meta-analysis and trial sequential analysis

**DOI:** 10.1186/s13643-019-1155-9

**Published:** 2019-10-28

**Authors:** Ulver Spangsberg Lorenzen, Katrine Bredahl Buggeskov, Emil Eik Nielsen, Naqash Javaid Sethi, Christian Lildal Carranza, Christian Gluud, Janus Christian Jakobsen

**Affiliations:** 1 Department of Vascular Surgery, Rigshospitalet, Copenhagen University Hospital, Copenhagen, Denmark; 2 Department of Thoracic Anaesthesiology, Rigshospitalet, Copenhagen University Hospital, Copenhagen, Denmark; 3 Copenhagen Trial Unit, Centre for Clinical Intervention Research, Department 7812, Rigshospitalet, Copenhagen University Hospital, Copenhagen, Denmark; 4 Department of Cardiothoracic Surgery, Rigshospitalet, Copenhagen University Hospital, Copenhagen, Denmark; 5 Copenhagen Trial Unit, Centre for Clinical Intervention Research, Department 7812, Rigshospitalet, Copenhagen University Hospital, Copenhagen, Denmark; 6 Department of Cardiology, Holbæk Hospital, Holbæk, Denmark; 7 Department of Regional Health Research, The Faculty of Health Sciences, University of Southern Denmark, Odense, Denmark

**Keywords:** Coronary artery bypass grafting, Ischaemic heart disease, Systematic review, Meta-analysis, Trial sequential analysis

## Abstract

**Background:**

Despite increasing survival, cardiovascular disease remains the primary cause of death worldwide with an estimated 7.4 million annual deaths. The main symptom of ischaemic heart disease is chest pain (angina pectoris) most often caused by blockage of a coronary artery. The aim of coronary artery bypass surgery is revascularisation achieved by surgically grafting harvested arteries or veins distal to the coronary lesion restoring blood flow to the heart muscle. Older evidence suggested a clear survival benefit of coronary artery bypass graft surgery, but more recent trials yield less clear evidence. We want to assess the benefits and harms of coronary artery bypass surgery combined with different medical therapies versus medical therapy alone in patients with ischaemic heart disease.

**Methods:**

This protocol for a systematic review follows the recommendations of Cochrane and the eight-step assessment procedure suggested by Jakobsen and colleagues. We plan to include all randomised clinical trials assessing coronary artery bypass surgery combined with different medical therapies versus medical therapy alone in patients with ischaemic heart disease. We plan to search the Cochrane Central Register of Controlled Trials (CENTRAL), MEDLINE, EMBASE, LILACS, Science Citation Index Expanded on Web of Science, and BIOSIS to identify relevant trials. Any eligible trial will be assessed as high risk or low risk of bias, and our conclusions will primarily be based on trials at low risk of bias. The analyses of the extracted data will be performed using Review Manager 5, STATA 16 and trial sequential analysis. For both our primary and secondary outcomes, we will create a ‘Summary of Findings’ table and use GRADE to assess the certainty of the evidence.

**Discussion:**

Coronary artery bypass surgery is invasive and can cause death, which is why its use must be thoroughly studied to determine if it yields a large enough long-term benefit for the thousands of patients receiving it every year.

**Systematic review registration:**

PROSPERO ID 131924

## Background

### Description of the condition

Ischaemic heart disease was first described in the eighteenth century by Giovanni Batti Morgagni and Friedrich Hoffman, who identified anginal syndromes and reduced coronary blood flow, respectively [1]. Ischaemic heart disease is caused by a mismatch between myocardial oxygen supply and demand. The pathophysiological pathways leading to this mismatch are (1) plaque-related obstruction of epicardial arteries, (2) focal or diffuse spasms of arteries (either normal or plaque-diseased), (3) microvascular dysfunction, and (4) left ventricular dysfunction caused by previous myocardial infarction and hibernation (i.e. myocardium with reduced metabolic demand) [2, 3]. Risk factors for atherosclerosis include smoking, hypertension, diabetes, hypercholesterolaemia, male sex, family disposition, and age [4].

The main symptom of ischaemic heart disease is chest pain (named angina pectoris when originating from ischaemic heart disease) most often caused by blockage of a coronary artery. Other symptoms, or ‘angina equivalents’, include shortness of breath, nausea, sweating, and fatigue [5]. Despite increasing survival [6], cardiovascular disease remains the primary cause of death worldwide with an estimated 7.4 million annual deaths [7]. Patients with ischaemic heart disease are also at risk of acquiring disabilities accounting for 5.2% of all disease-adjusted life years worldwide [8].

Ischaemic heart disease is diagnosed and subdivided by a combination of patient history, electrocardiograms, echocardiograms, stress testing, and blood measurement of myocardial enzymes [9]. Ischaemic heart disease may either be classified as stable ischaemic heart disease or acute coronary syndrome [2].

Stable ischaemic heart disease is defined as episodes of reversible myocardial hypoxia with none to minimal symptoms at rest and angina pectoris provoked by physical activities such as walking, emotion, or stress. There is spontaneous relief when ceasing physical stress or the administration of nitroglycerine [2].

Acute coronary syndrome is a common term for either unstable angina (pain without physical exertion), acute myocardial infarction defined by two of three criteria: angina pectoris and/or relevant electrocardiogram (ECG) changes (with or without ST-elevation) and/or blood measurement of myocardial ischaemic enzymes, and sudden cardiac death [9].

### Description of the intervention

The first coronary artery bypass graft surgeries were performed by Alexis Carrel on canines in 1910, using brachiocephalic and carotid arteries. The first human bypass graft surgery was performed by Arthur Vinberg in 1945, using the ‘Vinberg procedure’ by implanting the internal thoracic artery directly into the myocardium [10].

The aim of coronary artery bypass surgery is revascularisation achieved by surgically grafting harvested arteries or veins distal to the coronary lesion restoring blood flow to the heart muscle. Often, the revascularisation is achieved by connecting the left internal mammary artery directly to the left anterior descending artery or by using the saphenous vein or radial artery to bypass the blocked coronary artery connecting it with the aorta [10, 11]. The procedure is performed as open or less common minimal invasive heart surgery with access to the heart achieved through a median sternotomy or several small thoracic incisions, respectively [10, 12]. To ensure good surgical conditions, a still and blood empty heart is achieved by cardioplegia followed by extracorporal circulation in the form of cardiopulmonary bypass [14]. This surgical procedure is named coronary artery bypass grafting (CABG). In some patients, coronary artery bypass graft surgery is performed without the use of cardiopulmonary bypass (off-pump coronary artery bypass grafting, OPCAB), which accounted for approximately 17% in 2012 (after a steady decline since peak usage in 2002, likely due to the increased difficulty of the procedure and trials showing no clear benefit [13–15]) of all coronary artery bypass graft surgeries worldwide [13–16]. Recent technical advancements include endoscopic and no-touch technique saphenous vein harvesting, sequential grafting, storing grafts in buffered solution, and externally stented vein grafts [17, 18]. Also, newer types of coronary artery bypass surgery are evolving, including minimal invasive (endoscopic technique without median sternotomy), robotically assisted, and hybrid (percutaneous and off-pump) approaches, but they are still held back by their technical difficulties and somewhat disappointing preliminary results [11].

The primary choice of graft vessel is currently the left internal thoracic artery, which is usually grafted in situ to for example the left anterior descending artery [19]. Grafts are also obtained from the saphenous vein, radial artery, right internal thoracic artery, or gastroepiploic artery [19–22]. When using bilateral internal thoracic artery grafts, it is recommended to at least consider harvest of the arteries in a skeletonised (without surrounding tissue) fashion to reduce the risk of sternal wound complications, otherwise associated with bilateral grafting [13]. A recent trial showed no advantage comparing bilateral internal-thoracic-artery grafts versus single internal-thoracic-artery grafting in death from any cause at 10 years in the intention-to-treat analysis [24], but more evidence is needed.

According to American College of Cardiology Foundation and American Heart Association (ACCF/AHA) guidelines, coronary artery bypass surgery is the recommended intervention in patients with (1) three-vessel coronary disease; (2) stenosis of the proximal left anterior descending artery combined with > 70% stenosis of another coronary vessel; (3) left main coronary artery stenosis of > 50% of its diameter with an anatomy unfavourable for percutaneous coronary intervention (blockage of the coronary arteries is significant but disease is diffuse); and (4) ST-elevated myocardial infarction caused by a left main coronary artery stenosis of > 50% of its diameter, as long as the choice of coronary artery bypass does not improperly delay the treatment [25]. European Society of Cardiology and European Association for Cardio-Thoracic Surgery (ESC/EACTS) guidelines recommend coronary artery bypass surgery in patients with (1) proximal left anterior descending coronary artery disease, (2) left main coronary artery stenosis, and (3) three-vessel disease, especially at high SYNTAX scores [26].

The ‘heart team’ approach is usually used to choose between coronary artery bypass surgery, percutaneous coronary intervention, and medical therapy [27]. Using this approach, a cardiac surgeon, an interventionist cardiologist, and a general cardiologist determine the preferred strategy for the individual patient. The choice of treatment is generally made according to guidelines depending on the location of stenosis and the amount of coronary vessels affected (usually one, two or three), with a higher number signifying more advanced disease more likely to be treated with interventions like coronary artery bypass surgery or percutaneous coronary intervention [23, 24].

### How the intervention might work

Coronary artery bypass surgery aims towards revascularisation distally of a stenosis, resulting in restoration of blood flow to the myocardium [28]. This is thought to limit ischaemia and potentially infarct size, which in turn might relieve angina and benefit the patient with regards to mortality and re-infarction. As well, this could likely re-establish perfusion of areas of hibernating myocardium, likely protecting against further infarction.

The medical therapy (anticipated to be equal in both groups) that are indicated for ischaemic heart disease include anti-anginal therapies (e.g. beta-blockers, calcium-antagonist, short-acting nitrates and long-acting nitrates), cholesterol-reducing therapies (e.g. statins, fibrates and absorption inhibitors), antiplatelet therapies (e.g. aspirin or clopidogrel), and anticoagulation therapies (non-vitamin K oral antagonists and vitamin K antagonists). Anti-anginal therapies are thought to limit peripheral vascular resistance and thus reduce the required pressure the myocardium must create to ensure perfusion. Cholesterol-reducing therapies are thought to reduce the risk of plaque generation and development. Antiplatelet and anticoagulation therapies are thought to reduce the risk of thrombosis, usually related to plaque rupture.

### Why it is important to do this review

Early evidence between 1970 and 1980 suggested that coronary artery bypass surgery compared with medical therapy led to a survival benefit [27–31]. In 1994, a meta-analysis of seven randomised clinical trials found a survival benefit [32]. A randomised clinical trial from 2004 also indicated that coronary artery bypass surgery decreased cardiac death, myocardial infarction, and future revascularisations [33]. A recent Bayesian network meta-analysis found a survival benefit of coronary artery bypass surgery compared with medical therapy; however, when restricting the analysis to newer studies, the benefit was not statistically significant [34].

Revascularisation (combined with different medical therapies) compared with medical therapy has been reviewed before [32, 33], but since then, more information has been published. Recently, the 10-year results of the STITCH randomised clinical trial with 1212 participants compared the effects of coronary artery bypass surgery versus medical therapy in patients with ischaemic heart disease and heart failure. The results showed that coronary artery bypass surgery was associated with a decreased cardiovascular mortality and all-cause mortality [35]. Contrary to this result, a recent trial randomising diabetic participants with coronary artery disease found no increased survival of coronary artery bypass surgery versus medical therapy [36]. The International Study of Comparative Health Effectiveness with Medical and Invasive Approaches (ISCHEMIA) trial is currently underway [37].

The prevalence of ischaemic heart disease is considerable. One-third of all deaths in individuals over the age of 35 years are caused by ischaemic heart disease [38]. A considerable disease burden and healthcare cost can therefore be alleviated by beneficial treatments. We have not been able to identify any updated systematic review assessing the effects of coronary artery bypass surgery versus medical therapy for ischaemic heart disease taking into account risks of random errors, design errors, and systematic errors [38, 39]. There is therefore an urgent need for a systematic review assessing the effects of coronary artery bypass surgery versus medical therapy for ischaemic heart disease.

## Objectives

The aim of this study is to assess the benefits and harms of coronary artery bypass surgery combined with different medical therapies versus medical therapy alone in patients with ischaemic heart disease.

## Methods

### Criteria for considering studies for this review

#### Types of studies

We will only include randomised clinical trials, irrespective of publication type, reported outcomes, publication status, publication date, and language for assessments of benefits and harms. During the selection of trials, we will include observational studies (i.e. quasi-randomised studies, cohort studies, or patient reports) that reported adverse events caused by or associated with coronary artery bypass surgery combined with different medical therapies. However, we will not specifically search for such observational studies for inclusion in this review, which is a known limitation of our systematic review. If benefits of coronary artery bypass surgery combined with different medical therapies are found, then systematic reviews of harms based on observational studies need to be conducted [41].

#### Types of participants

We will include any adult participant (≥ 18 years old) diagnosed with ischaemic heart disease, that is, acute myocardial infarction (with or without ST-elevation), previous myocardial infarction, unstable angina, or stable angina. We will accept the definitions used by the individual trialists.

In the case of trials, where only a part of the participants fulfils our inclusion criteria, we will include the data on the part fulfilling the criteria. If the data are not clearly separated, we will try to obtain the data from trialists.

#### Types of interventions

The experimental intervention will be any type of coronary artery bypass surgery (as defined by trialists) combined with medical therapies (please see below).

The control intervention will be any type of medical therapy indicated for ischaemic heart disease, such as anti-anginal therapies (e.g. beta-blockers, calcium-antagonist, short-acting nitrates and long-acting nitrates), cholesterol-reducing therapies (e.g. statins, fibrates and absorption inhibitors), antiplatelet therapies (e.g. aspirin or clopidogrel), and anticoagulation therapies (non-vitamin K oral antagonists and vitamin K antagonists).

We will accept any type of co-intervention when such co-intervention is delivered similarly in both intervention groups. We will assume that the effects of the co-interventions will ‘even out’ in both groups so that the possible effects of either intervention will be reflected in the results.

### Types of outcome measures

#### Primary outcomes

1. All-cause mortality.
2. Proportion of participants with one or more serious adverse events. We will define a serious adverse event as any untoward medical occurrence that resulted in death, was life threatening, was persistent, led to significant disability, required hospitalisation or prolonged existing hospitalisation or jeopardised the participant, according to ICH Good Clinical Practice Guidelines (ICH-GCP) [42]. As we expect the reporting of serious adverse events to be very heterogeneous and not strictly according to the ICH-GCP recommendation in many trials, we will also include an event as a serious adverse event if the trialists either (1) use the term ‘serious adverse event’ but do not refer to ICH-GCP, or (2) report the proportion of participants with an event we consider fulfils the ICH-GCP definition. If several of such events are reported, then we will choose the highest proportion reported in each trial.
3. Quality of life measured on any valid scale, such as SF-36 [43].

#### Secondary outcomes

1. Cardiovascular mortality (as defined by trialists).
2. Myocardial infarction (as defined by trialists).
3. Stroke, as defined by trialist, e.g. computed tomography (CT)-verified cerebral infarction or relevant neurological defects.
4. Angina on a continuous scale such as Seattle Angina Questionnaire [44].
5. Proportion of participants with one or more adverse events considered to be non-serious.

#### Exploratory outcomes

1. Proportion of participants with individual serious adverse events.
2. Proportion of participants with individual adverse events considered to be non-serious.
3. Number of serious adverse events (analysed as count data).

We will assess all outcomes at maximal follow-up.

### Search methods for identification of studies

#### Electronic searches

We will identify trials through systematic searches of the following bibliographic databases:

- Cochrane Central Register of Controlled Trials (CENTRAL) in The Cochrane Library.
- MEDLINE (Ovid, from 1946 onwards).
- Embase (Ovid, from 1980 onwards).
- Web of Science Core Collection (Thomson Reuters, from 1900 onwards)
- BIOSIS (Thomson Reuters, from 1926 onwards)
- LILACS (from 1982 onwards)
- CINAHL (from 1937 onwards)
- SCOPUS (from 1966 onwards)

The preliminary search strategy for MEDLINE (Ovid) will be adapted for use in the other databases. The Cochrane sensitivity-maximising randomised clinical trial filter [45] will be applied to MEDLINE (Ovid) and adaptations of it to the other databases, except CENTRAL. We will search the files of the FDA (www.fda.gov) and the EMA (http://www.ema.europa.eu) regulatory authorities. We will also conduct a search of ClinicalTrials.gov (www.ClinicalTrials.gov) and the WHO International Clinical Trials Registry Platform (ICTRP) Search Portal (http://apps.who.int/trialsearch/) for on-going or unpublished trials. We will also inquire the Australian-New Zealand Food and Drug Administration, Chinese Food and Drug Administration, Japanese Food and Drug Administration and Russian Food and Drug Administration about any trials they may know of.

We will search all databases from their inception to the present, and we will impose no restriction on language of publication or publication status.

We will not perform a separate search for adverse effects of interventions used for the treatment of ischaemic heart disease. We will consider adverse effects described in included studies only.

#### Searching other resources

We will check reference lists of all included studies and any relevant systematic reviews identified for additional references to trials. We will also examine any relevant retraction statements and errata for included studies.

### Data collection and analysis

We will perform the review using Review Manager 5 and Trial Sequential Analysis. In case of Review Manager statistical software not being sufficient, we will use STATA.

#### Selection of studies

Two review authors (USL, KBB) will independently screen titles and abstracts for inclusion of all potential studies we identify as a result of the search and code them as ‘retrieve’ (eligible or potentially eligible/unclear) or ‘do not retrieve’. If there are any disagreements, a third author will be asked to arbitrate (JCJ). We will retrieve the full-text study reports/publication and two review authors (USL, KBB) will independently screen the full-text and identify studies for inclusion and identify and record reasons for exclusion of the ineligible studies. We will resolve any disagreement through discussion or, if required, we will consult a third person (JCJ). We will identify and exclude duplicates and collate multiple reports of the same study so that each study rather than each report is the unit of interest in the review. We will record the selection process in sufficient detail to complete a PRISMA flow diagram and ‘Characteristics of excluded studies’ table.

#### Data extraction and management

We will use a data collection form for study characteristics and outcome data, which has been piloted on at least one study in the review. Two review authors (USL, KBB) will extract study characteristics from included trials. We will extract the following study characteristics.

1. Methods: study design, total duration of study, details of any ‘run in’ period, number of study centres and location, study setting, method of withdrawal, and date of study.
2. Participants: number randomised, number lost to follow-up/withdrawn, number analysed, mean age, age range, sex, severity of condition, diagnostic criteria, inclusion criteria, exclusion criteria, and baseline criteria (such as number of patients with hypertension, diabetes, lung disease, etc.).
3. Interventions: intervention, comparison, concomitant medications, excluded medications, and the use of coronary stents in both control and intervention group.
4. Outcomes: primary, secondary, and exploratory outcomes specified and collected, and time points reported, differences in planned and reported outcomes.
5. Notes: funding for trial, and notable conflicts of interest of trial authors.

Two review authors (USL, KBB) will independently extract outcome data from included studies. We will resolve disagreements by consensus or by involving a third person (JCJ). One review author (USL) will transfer data into the Review Manager (RevMan 2014) file. We will double-check that data is entered correctly by comparing the data presented in the systematic review with the study reports. A second review author (KBB) will spot-check study characteristics for accuracy against the trial report.

#### Assessment of risk of bias in included studies

Two review authors (USL, KBB) will independently assess risk of bias for each study using the criteria outlined in the *Cochrane Handbook for Systematic Reviews of Interventions* [46]. We will resolve any disagreements by discussion or by involving another author (JCJ). We will assess the risk of bias according to the following domains.

1. Random sequence generation.
2. Allocation concealment.
3. Blinding of participants and personnel.
4. Blinding of outcome assessment.
5. Incomplete outcome data.
6. Selective outcome reporting.
7. Other bias.

We will grade each domain for potential risk of bias as high, low or unclear and provide a quote from the study report together with a justification for our judgment in the ‘Risk of bias’ table. We will summarise the risk of bias judgements across different studies for each of the domains listed. Where information on risk of bias relates to unpublished data or correspondence with a trialist, we will note this in the ‘Risk of bias’ table.

This enables us to classify each trial and each outcome at low risk of bias and at high risk of bias. The trials at unclear risk of bias and at high risk of bias tend to overestimate benefits and underestimate harms and are therefore considered trials at overall high risk of bias (see below) [46–52].

### Bias domains

#### Random sequence generation

- Low risk of bias: sequence generation was achieved using computer random number generation or a random number table. Drawing lots, tossing a coin, shuffling cards and throwing dice were adequate if performed by an independent person not otherwise involved in the trial.
- Unclear risk of bias: the method of sequence generation was not specified.
- High risk of bias: the sequence generation method was not random or only quasi-randomised.

#### Allocation concealment

- Low risk of bias: the allocation sequence was described as unknown to the investigators. Hence, the participants’ allocations could not have been foreseen in advance of, or during, enrolment. Allocation was controlled by a central and independent randomisation unit, an on-site locked computer, identical looking numbered sealed opaque envelopes, drug bottles or containers prepared by an independent pharmacist, or an independent investigator.
- Unclear risk of bias: it was unclear if the allocation was hidden or if the block size was relatively small and fixed so that intervention allocations may have been foreseen in advance of, or during, enrolment.
- High risk of bias: the allocation sequence was likely to be known to the investigators who assigned the participants.

#### Blinding of participants and treatment providers

- Low risk of bias: it was described that both participants and treatment providers were blinded to treatment allocation.
- Unclear risk of bias: it was unclear whether participants and treatment providers were blinded, or the extent of blinding was insufficiently described.
- High risk of bias: no blinding or incomplete blinding of participants and treatment providers was performed.

We do not expect to identify any trials using adequate blinding of participants and personnel due to the nature of the coronary artery bypass surgery. Hence, we will not use this bias domain when assessing the 'overall risk of bias'.

#### Blinding of outcome assessment

- Low risk of bias: it was mentioned that outcome assessors were blinded and this was described.
- Unclear risk of bias: it was not mentioned whether the outcome assessors were blinded, or the extent of blinding was insufficiently described.
- High risk of bias: no blinding or incomplete blinding of outcome assessors was performed.

#### Incomplete outcome data

- Low risk of bias: missing data were unlikely to make intervention effects depart from plausible values. This could either be (1) there were no drop-outs or withdrawals; or (2) the numbers and reasons for the withdrawals and drop-outs for all outcomes were clearly stated and could be described as being similar in both groups, and the trial handled missing data appropriately in an intention-to-treat analysis using proper methods (e.g. multiple imputations). Generally, the trial was judged to be at a low risk of bias due to incomplete outcome data if drop-outs were less than 5%. However, the 5% cut-off was not definitive.
- Unclear risk of bias: there was insufficient information to assess whether missing data were likely to induce bias on the results.
- High risk of bias: the results were likely to be biased due to missing data either because the pattern of drop-outs could be described as being different in the two intervention groups or the trial used improper methods in dealing with the missing data (e.g. last observation carried forward).

#### Selective outcome reporting

- Low risk of bias: a protocol was published before randomisation began and all outcome results were reported adequately. If there is no protocol or the protocol is published after the trial has begun, reporting of all-cause mortality and various types of serious adverse events will grant the trial a grade of ‘low risk of bias’ for this bias domain.
- Unclear risk of bias: no protocol was published.
- High risk of bias: the outcomes in the protocol were not reported on.

#### Other bias

- Low risk of bias: the trial appeared to be free of other bias domains that could put it at risk of bias.
- Unclear risk of bias: the trial may or may not have been free of other domains that could put it at risk of bias.
- High risk of bias: there were other factors in the trial that could put it at risk of bias.

#### Overall risk of bias

We will judge trials to be at an ‘overall low risk of bias’ if they are assessed as ‘low risk of bias’ in all the above domains regardless of the assessment of 'blinding of participants and treatment providers’. We will judge trials to be at an ‘overall high risk of bias’ if they are assessed as having unclear risk of bias or high risk of bias in one or more of the above domains.

We will assess the domains ‘Blinding of outcome assessment’, ‘Incomplete outcome data’ and ‘Selective outcome reporting’ for each outcome result. Thus, we will assess the bias risk for each outcome result in addition to the overall bias risk for each trial.

For additional details on how we will assess the risk of bias in included studies, see Additional file 1.

### Assessment of bias in conducting the systematic review

We will conduct the review according to this published protocol and report any deviations from it in the ‘Differences between protocol and review’ section of the systematic review.

#### Measures of treatment effect

We will analyse dichotomous data as risk ratios with 95% confidence intervals*,* as well as the trial sequential analysis-adjusted confidence intervals, and continuous data as mean difference or standardised mean difference with 95% confidence intervals*,* as well as the trial sequential analysis-adjusted confidence intervals. We will enter data presented as a scale with a consistent direction of effect. We will use the standardised mean difference (SMD) when the trials all assess the same outcome but measure it in a variety of ways, for example, they use different scales [54]. Regarding count data, we will calculate rate ratios with 95% confidence intervals.

We will narratively describe skewed data reported as medians and interquartile ranges.

#### Unit of analysis issues

We will only include randomised clinical trials so we will have no unit of analysis issues.

For cross-over trials, we will only include participants from the first treatment period in the trial [54, 55]. We will avoid counting data more than once from participants in control arms of trials with multiple experimental intervention arms by dividing the sample size and number of participants experiencing the event by the number of eligible treatment arms used.

For cluster randomised trials, we will downgrade their finding with their intracluster correlation coefficient and make subgroup analysis comparing individually randomised trials to cluster randomised trials.

#### Dealing with missing data

We will contact investigators or study sponsors in order to verify key trial characteristics and obtain missing numerical outcome data where possible (e.g. when a study is identified as abstract only). Where this is not possible, and the missing data are thought to introduce serious bias, we will explore the impact of including such studies in the overall assessment of results by a sensitivity analysis (see ‘Sensitivity analysis’ section).

### Dichotomous outcomes

We will not use intention-to-treat data if the original report did not contain them. We will not impute missing values for any outcomes in our primary analysis.

### Continuous outcomes

If both end scores and change from baseline scores are reported then we will use end scores. If only change values are reported, the results will be analysed together with end scores [56]. If standard deviations are not reported, we will calculate them using data from the trial if possible. We will not use intention-to-treat data if the original report did not contain such data. We will not impute missing values for any outcomes in our primary analysis.

In our sensitivity analysis for dichotomous and continuous outcomes, we will impute data; see ‘Sensitivity analysis’ section.

#### Assessment of heterogeneity

We will primarily visually investigate forest plots to assess heterogeneity. We will secondly assess the presence of statistical heterogeneity by the Chi^2^ test (threshold *P* < 0.10) and measure the quantities of heterogeneity using the *I*^2^ statistic [57, 58].

If we identify substantial heterogeneity, we will report it and explore possible causes by prespecified subgroup analysis, and we will ultimately decide if meta-analysis is appropriate [54].

#### Assessment of reporting biases

If we are able to pool more than ten trials, we will create and examine a funnel plot to explore possible small study biases for the primary outcomes. Using the asymmetry of the funnel plot, we will assess the risk of bias. For dichotomous outcomes, we will test asymmetry with the Harbord test [59]. For continuous outcomes, we will use the regression asymmetry test [60] and the adjusted rank correlation [61].

#### Data synthesis

##### Meta-analysis

Our methodology is based on the recommendations stated in the *Cochrane Handbook for Systematic Reviews of Interventions* [62], according to Keus and colleagues [40], and according to the eight-step assessment suggested by Jakobsen and colleagues [39]. We will use the statistical software Review Manager 5 and STATA 16 to meta-analyse data. We will in a sensitivity analysis compare trials with low risk of bias with all trials, and if these results do not differ our conclusions will be based on all trials. Otherwise, we will base our primary conclusions on the results of the analyses of the outcomes with a low risk of bias in all bias risk domains. The limitations of the expected lack of ‘blinding of participants and personnel’ for conclusions will be thoroughly discussed in the final review [63, 64].

##### Trial sequential analysis

Cumulative meta-analyses are at risk of producing random errors due to sparse data and multiple testing of accumulating data [56, 65–69]; therefore, trial sequential analysis [70, 71] can be applied to control this risk (http://www.ctu.dk/tsa/) [72]. The required information size (that is the number of participants needed in a meta-analysis to detect or reject a certain intervention effect) can be calculated in order to minimise random errors [69, 73]. The required information size takes into account the event proportion in the control group, the assumption of a plausible relative risk (RR) reduction and the heterogeneity of the meta-analysis [69, 73, 74]. Trial sequential analysis enables testing for significance to be conducted each time a new trial is included in the meta-analysis. On the basis of the required information size, trial sequential monitoring boundaries can be constructed. This enables one to determine the statistical inference concerning cumulative meta-analysis that has not yet reached the required information size [69].

Firm evidence for benefit or harm may be established if the trial sequential monitoring boundary is crossed before reaching the required information size, in which case further trials may turn out to be superfluous. In contrast, if the boundaries for benefit or harm are not surpassed, we may conclude that it is necessary to continue with further trials before a certain intervention effect can be detected or rejected. Firm evidence for lack of the postulated intervention effect can also be assessed with trial sequential analysis. This occurs when the cumulative Z-score crosses the trial sequential monitoring boundaries for futility.

To control the risks of random error, we have used relatively conservative estimations of the anticipated intervention effect estimates [39]. Large anticipated intervention effects lead to small required information sizes and the thresholds for significance will be less strict after the information size has been reached [39].

We will analyse all primary and secondary outcomes with trial sequential analysis. Through these analyses, we will calculate the trial sequential analysis-adjusted confidence intervals. We will use the following assumptions:

##### Primary outcomes

We will estimate the diversity adjusted required information size [73] based on the observed proportion of patients with an outcome in the control group. We have three primary outcomes. Therefore, we will use an alpha of 2.5% [39], a beta of 10%, and the diversity suggested by the trials in the meta-analysis [39]. As anticipated intervention effects for the primary outcomes in the Trial Sequential Analysis, we will use the following relative risk reductions or increases, because these seem to be the maximum realistic intervention effect estimates based on former studies, trials, and meta-analyses:

- All-cause mortality: relative risk reduction of 15% [29, 32, 34, 35].
- Serious adverse events: relative risk reduction of 20%.
- Quality of life: we will use the observed standard deviation and a clinically-relevant mean difference equal to standard deviation/2.

##### Secondary outcomes

We will estimate the diversity adjusted required information size [73] based on the proportion of patients with an outcome in the control group. We have five secondary outcomes. Therefore, we will use an alpha of 1.7% [39], a beta of 10%, and the diversity suggested by the trials in the meta-analysis [39].

As anticipated intervention effects for the secondary outcomes in the Trial Sequential Analysis, we will use the following relative risk reductions or increases because they seem to be realistic intervention effect estimates based on former studies, trials and meta-analyses as cited below:

- Cardiovascular death: relative risk reduction of 20%.
- Angina: we will use the observed standard deviation, a clinically-relevant mean difference equal to standard deviation/2.
- Stroke: relative risk reduction of 20%
- Myocardial infarction: relative risk reduction of 20% [29, 32].

We will additionally include age as a covariate in meta-regression to assess whether age influences the effect of coronary artery bypass graft surgery.

##### Assessment of significance

We will assess our intervention effects with both random-effects [75] and fixed-effect meta-analyses [76], and we will use the more conservative point estimate of the two [39]. The more conservative point estimate is the estimate closest to zero effect. If the two estimates are equal, the estimate with the widest confidence interval will be used. In the case that few trials (1–3) make up > 95% of the weight in the meta-analysis, we will use fixed-effects meta-analysis. We assess three primary outcomes and for the primary outcomes, we will therefore consider a *P* value less than *P* < 0.025 as significant [39] in order to preserve the family wise error rate below 0.05. We assess five secondary outcomes and will hence consider a *P* value less than *P* < 0.017 as significant. For our exploratory outcomes, we will consider a *P* value less than *P* < 0.05 as significant. We will use the eight-step procedure to assess whether the thresholds for significance are crossed or not [39].

#### Subgroup analysis and investigation of heterogeneity

We will perform the following subgroup analyses on all our primary outcomes and secondary outcomes comparing the effect of coronary artery bypass surgery versus medical therapy between trials:

1. Trials at low risk of bias compared to trials at high risk of bias.
2. Trials at low risk of bias compared to trials at high risk of bias ignoring blinding of patients and personnel.
3. According to comparability of medical intervention in the surgery groups and control groups.
4. According to which conduits were used in the surgical group:
  - Left (and right) internal mammarial artery grafts
  - Saphenous vein grafts
  - Radial artery grafts
  - Other grafts (e.g. gastroepiploic grafts)
5. According to severity of indication:
  - One-vessel disease
  - Two-vessel disease
  - Three-vessel disease
6. According to trials on elective procedures (often patients with stable ischaemic heart disease) compared to trials with acute and sub-acute procedures (often patients with NSTEMI and unstable angina pectoris).
7. According to length of maximal follow-up:
  - Up to 1 year
  - 1 year up to and including 5 years
  - More than 5 years
8. According to trial publication dates (before 2000 compared to 2000 or later).
9. According to patient ejection above compared to at or below 45% (significant left ventricular systolic heart failure).
10. According to absence or presence of diabetes mellitus.

We will use the formal test for subgroup interactions in Review Manager (RevMan 2014) and STATA 16.

#### Sensitivity analysis

To assess the potential impact of bias, we will perform a sensitivity analysis in which we exclude trials with overall ‘high risk of bias’ in all risk of bias domains.

To assess the potential impact of the missing data, we will perform the following analyses:

1. ‘Best-worst-case’ scenario: It will be assumed that all participants lost to follow up in the experimental group survived, had no serious adverse event, had no myocardial infarction, had a beneficial outcome in quality of life, had a beneficial outcome in level of angina, had no stroke, and had no non-serious adverse event; while all those with missing outcomes in the control group have not survived, had a serious adverse event, had a myocardial infarction, had a harmful outcome in quality of life, had a harmful outcome in level of angina, had a stroke, and had a non-serious adverse event.
2. ‘Worst-best-case’ scenario: It will be assumed that all participants lost to follow up in the experimental group did not survive, had a serious adverse event, had a myocardial infarction, had a harmful outcome in quality of life, had a harmful outcome in level of angina, had a stroke, and had a non-serious adverse events; while all those with missing outcomes in the control group survived, had no serious adverse event, had no myocardial infarction, had a beneficial outcome in quality of life, had a beneficial outcome in level of angina, had no stroke, and had no non-serious adverse event.

Results from both scenarios will be presented in our review. When analysing continuous outcomes regarding missing data, a ‘beneficial outcome’ will be the group mean plus two standard deviations (and one standard deviation) of the group mean, and a ‘harmful outcome’ will be the group mean minus two standard deviations (and one standard deviation) of the group mean [39].

To assess the potential impact of missing standard deviations for continuous outcomes, we will perform the following sensitivity analyses:

1. Where standard deviations are missing and not possible to calculate, we will impute them from trials with similar populations and low risk of bias. If no such trials can be found, we will impute standard deviations from trials with a similar population. As the final option, we will impute standard deviations from all trials.

##### Summary of findings table

We will create a summary of findings table using the following outcomes: all-cause mortality, serious adverse events, cardiovascular mortality, myocardial infarction, angina, quality of life, stroke, and non-serious adverse events*.* We will use the five GRADE considerations (study limitations, consistency of effect, imprecision, indirectness, and publication bias) to assess the certainty of a body of evidence as it relates to the studies which contribute data to the meta-analyses for the prespecified outcomes. We will also use trial sequential analysis to assess ‘imprecision’ [39]. Otherwise, we will use methods and recommendations described in Section 8.5 and Chapter 12 of the *Cochrane Handbook for Systematic Reviews of Interventions* [62] using GRADEpro software (https://gradepro.org/). We will justify all decisions to downgrade the quality of studies using footnotes and we will make comments to aid reader’s understanding of the review where necessary.

Judgements about certainty of the body of evidence will be made by two review authors (USL, KBB) working independently, with disagreements resolved by discussion or involving a third author (JCJ). Judgements will be justified, documented and incorporated into reporting of results for each outcome.

We plan to extract study data, format our comparisons in data tables and prepare a summary of findings table before writing the results and conclusions of our review. A template summary of findings table is included as Table 1.

**Table 1:** Summary of findings template

Coronary artery bypass surgery compared with medical therapy alone for ischaemic heart disease					
Patient or population: Adult patients with ischaemic heart disease Settings: Hospital Intervention: Coronary artery bypass surgery Comparison: Medical therapy					
**Outcomes**	**Illustrative comparative risks** ^**a**^ **(95% CI)**		**Relative effect (95% CI)**	**No of Participants (studies)**	**Quality of the evidence (GRADE)**
	**Assumed risk**	**Corresponding risk**			
	**Medical therapy**	**Coronary artery bypass surgery**			
All-cause mortality [follow-up]	Low risk population		RR [value] ([value] to [value])	[value] ([value])	⊕⊝⊝⊝ very low ⊕⊕⊝⊝ low ⊕⊕⊕⊝ moderate ⊕⊕⊕⊕ high
	[value] per 1000	[value] per 1000 ([value] to [value])			
	Medium risk population				
	[value] per 1000	[value] per 1000 ([value] to [value])			
	High risk population				
	[value] per 1000	[value] per 1000 ([value] to [value])			
Serious adverse events [follow-up]	Low risk population		RR [value] ([value] to [value])	[value] ([value])	⊕⊝⊝⊝ very low ⊕⊕⊝⊝ low ⊕⊕⊕⊝ moderate ⊕⊕⊕⊕ high
	[value] per 1000	[value] per 1000 ([value] to [value])			
	Medium risk population				
	[value] per 1000	[value] per 1000 ([value] to [value])			
	High risk population				
	[value] per 1000	[value] per 1000 ([value] to [value])			
Quality of life [follow-up]	The mean quality of life rating ranged across control groups from [value][measure]	The mean quality of life rating in the intervention groups was [value] [lower/higher] [(value to value lower/higher)]		[value] ([value])	
Cardiovascular mortality [follow-up]	Low risk population		RR [value] ([value] to [value])	[value] ([value])	⊕⊝⊝⊝ very low ⊕⊕⊝⊝ low ⊕⊕⊕⊝ moderate ⊕⊕⊕⊕ high
	[value] per 1000	[value] per 1000 ([value] to [value])			
	Medium risk population				
	[value] per 1000	[value] per 1000 ([value] to [value])			
	High risk population				
	[value] per 1000	[value] per 1000 ([value] to [value])			
Myocardial infarction [follow-up]	Low risk population		RR [value] ([value] to [value])	[value] ([value])	⊕⊝⊝⊝ very low ⊕⊕⊝⊝ low ⊕⊕⊕⊝ moderate ⊕⊕⊕⊕ high
	[value] per 1000	[value] per 1000 ([value] to [value])			
	Medium risk population				
	[value] per 1000	[value] per 1000 ([value] to [value])			
	High risk population				
	[value] per 1000	[value] per 1000 ([value] to [value])			
Angina [follow-up]	The mean angina rating ranged across control groups from [value][measure]	The mean angina rating in the intervention groups was [value] [lower/higher] [(value to value lower/higher)]		[value] ([value])	⊕⊝⊝⊝ very low ⊕⊕⊝⊝ low ⊕⊕⊕⊝ moderate ⊕⊕⊕⊕ high
Stroke [follow-up]	Low risk population		RR [value] ([value] to [value])	[value] ([value])	⊕⊝⊝⊝ very low ⊕⊕⊝⊝ low ⊕⊕⊕⊝ moderate ⊕⊕⊕⊕ high
	[value] per 1000	[value] per 1000 ([value] to [value])			
	Medium risk population				
	[value] per 1000	[value] per 1000 ([value] to [value])			
	High risk population				
	[value] per 1000	[value] per 1000 ([value] to [value])			
Non-serious adverse events [follow-up]	Low risk population		RR [value] ([value] to [value])	[value] ([value])	⊕⊝⊝⊝ very low ⊕⊕⊝⊝ low ⊕⊕⊕⊝ moderate ⊕⊕⊕⊕ high
	[value] per 1000	[value] per 1000 ([value] to [value])			
	Medium risk population				
	[value] per 1000	[value] per 1000 ([value] to [value])			
	High risk population				
	[value] per 1000	[value] per 1000 ([value] to [value])			

GRADE Working Group grades of evidence

High quality: further research is very unlikely to change our confidence in the estimate of effect. Moderate quality: Further research is likely to have an important impact on our confidence in the estimate of effect and may change the estimate. Low quality: Further research is very likely to have an important impact on our confidence in the estimate of effect and is likely to change the estimate. Very low quality: We are very uncertain about the estimate

^a^The basis for the assumed risk (e.g. the median control group risk across studies) is provided in footnotes. The corresponding risk (and its 95% confidence interval) is based on the assumed risk in the comparison group and the relative effect of the intervention (and its 95% CI)

*CI* confidence interval, *RR* risk ratio; other abbreviations, e.g. OR, etc.

Our primary summary of findings tables and conclusions will be based on the results of all trials, unless sensitivity analyses show a difference in the results of all trials versus trials with low risk of bias. In that case, we will base our primary conclusions on trials with low risk of bias. However, we do not expect to find any trials blinding participants and personnel, due to the nature of the procedure. Trials will be evaluated in all bias risk domains [47, 48, 51–53, 77, 78].

## Discussion

The purpose of this protocol for a systematic review is to compare the effects of coronary artery bypass graft surgery with the effects of medical treatment in patients with ischaemic heart disease. The outcomes will be all-cause mortality, serious adverse events, quality of life, cardiovascular death, myocardial infarction, stroke, angina, and adverse events considered to be non-serious. The surgery in question is invasive, which is why its use must be thoroughly studied to determine if it yields a large enough long-term benefit for the thousands of patients receiving it every year.

The strength of this protocol is firstly, that it compares interventions on a clinically relevant decision tree, allowing the results to be interpreted in relation to actual patients. Secondly, it utilises predefined methodology based on the Cochrane Handbook for Systematic Reviews of Interventions [62], Keus and colleagues’ matrix [40], the eight step assessment suggested by Jakobsen and colleagues [39], Trial Sequential Analysis [70], and GRADE assessments [79–81]. Thus, this protocol considers both risks of random errors, risks of design errors, and risks of systematic errors.

Our protocol also has limitations. Mainly, each strategy includes several treatments (e.g. the medical group is composed of various drugs), which could potentially complicate the interpretation of our results. It is the hope that our use of subgroup analyses will minimise this limitation. Conversely, doing multiple comparisons increase the risk of type 1 error. To attenuate this effect, we have adjusted our thresholds for significance in accordance with the number of primary outcomes included. However, our number of subgroups still create risk of type 1 error, which will be considered when interpreting the results of this review. Moreover, by design focusing primarily on randomised clinical trials, we will put more emphasis on potential benefits than on potential harms.

## Supplementary information

**Additional file 1.** Risk of bias assessment.

## Data Availability

The authors declare that the data supporting the findings of this study are available within the article and its supplementary information files.

## Abbreviations

ACCF/AHA: American College of Cardiology Foundation and American Heart Association
CABG: Coronary artery bypass grafting
CT: Computed tomography
ECG: Electrocardiogram
ESC/EACTS: European Society of Cardiology and European Association for Cardio-Thoracic Surgery
GCP: Good clinical practice
ICH: International Conference on Harmonisation of Technical Requirements for Registration of Pharmaceuticals for Human Use
ICH-GCP: ICH Good Clinical Practice Guidelines
ICTRP: International Clinical Trials Registry Platform
ISCHEMIA: International Study of Comparative Health Effectiveness with Medical and Invasive Approaches
OPCAB: Off-pump coronary artery bypass grafting
RR: Risk ratio
SF-36: Short Form 36
SMD: Standardised mean difference
WHO: World Health Organization
