# Supplementary material for: Coronary artery bypass surgery plus medical therapy versus medical therapy alone for ischaemic heart disease: a protocol for a systematic review with meta-analysis and trial sequential analysis
Source: Syst Rev. 2019 Oct 28;8:246. doi: 10.1186/s13643-019-1155-9 (PMC6819611; doi:10.1186/s13643-019-1155-9)
Supplement: Supplementary file 1 — Additional file 1. Risk of bias assessment. [file 13643_2019_1155_MOESM1_ESM.docx]

# Additional file 1: Risk of bias assessment

**Allocation sequence generation**

- Low risk of bias: sequence generation was achieved using computer random number generation or a random number table. Drawing lots, tossing a coin, shuffling cards, and throwing dice were adequate if performed by an independent person not otherwise involved in the trial.
- Unclear risk of bias: the method of sequence generation was not specified.
- High risk of bias: the sequence generation method was not random or only quasi-randomised.

**Allocation concealment**

- Low risk of bias: the allocation sequence was described as unknown to the investigators. Hence, the participants' allocations could not have been foreseen in advance of, or during, enrolment. Allocation was controlled by a central and independent randomisation unit, an on-site locked computer, identical looking numbered sealed opaque envelopes, drug bottles or containers prepared by an independent pharmacist, or an independent investigator.
- Unclear risk of bias: it was unclear if the allocation was hidden or if the block size was relatively small and fixed so that intervention allocations may have been foreseen in advance of, or during, enrolment.
- High risk of bias: the allocation sequence was likely to be known to the investigators who assigned the participants.

**Blinding of participants and treatment providers**

- Low risk of bias: it was described that both participants and treatment providers were blinded to treatment allocation.
- Unclear risk of bias: it was unclear whether participants and treatment providers were blinded, or the extent of blinding was insufficiently described.
- High risk of bias: no blinding or incomplete blinding of participants and treatment providers was performed.

We do not expect to identify any trials using adequate blinding of participants and personnel due to the nature of the coronary artery bypass surgery.

**Blinding of outcome assessment**

- Low risk of bias: it was mentioned that outcome assessors were blinded and this was described.
- Unclear risk of bias: it was not mentioned whether the outcome assessors were blinded, or the extent of blinding was insufficiently described.
- High risk of bias: no blinding or incomplete blinding of outcome assessors was performed.

**Incomplete outcome data**

- Low risk of bias: missing data were unlikely to make intervention effects depart from plausible values. This could either be: 1) there were no drop-outs or withdrawals; or 2) the numbers and reasons for the withdrawals and drop-outs for all outcomes were clearly stated and could be described as being similar in both groups, and the trial handled missing data appropriately in an intention-to-treat analysis using proper methods (e.g. multiple imputations). Generally, the trial was judged to be at a low risk of bias due to incomplete outcome data if drop-outs were less than 5%. However, the 5% cut-off was not definitive.
- Unclear risk of bias: there was insufficient information to assess whether missing data were likely to induce bias on the results.
- High risk of bias: the results were likely to be biased due to missing data either because the pattern of drop-outs could be described as being different in the two intervention groups or the trial used improper methods in dealing with the missing data (e.g. last observation carried forward).

**Selective outcome reporting**

- Low risk of bias: a protocol was published before randomisation began and all outcome results were reported adequately.
- Unclear risk of bias: no protocol was published.
- High risk of bias: the outcomes in the protocol were not reported on.

**Vested interest bias**

- Low risk of bias: it was described that the trial was not sponsored by any pharmaceutical company, any person, or any group with a financial or other interest in a certain result of the trial.
- Unclear risk of bias: it was unclear how the trial was sponsored.
- High risk of bias: the trial was sponsored by a pharmaceutical company, a person, or a group with a certain financial or other interest in a given result of the trial.

**Other bias**

- Low risk of bias: the trial appeared to be free of other bias domains that could put it at risk of bias.
- Unclear risk of bias: the trial may or may not have been free of other domains that could put it at risk of bias.
- High risk of bias: there were other factors in the trial that could put it at risk of bias.
